# *Bartonella quintana *coinfection with *Mycobacterium avium *complex and CMV in an AIDS patient: case presentation

**DOI:** 10.1186/1471-2334-6-89

**Published:** 2006-05-29

**Authors:** Clarisse Rovery, Jean Marc Rolain, Hubert Lepidi, Christine Zandotti, Jacques Moreau, Philippe Brouqui

**Affiliations:** 1 Service de maladies infectieuses et de médecine tropicale, hôpital Nord, APHM, Marseille, France; 2 Unité des Rickettsies, IFR 48, CNRS UMR 6020, Université de la Méditerranée, Faculté de médecine, Marseille, France; 3 Laboratoire de Microbiologie, Hôpital de la Timone, APHM, Marseille, France

## Abstract

**Background:**

As a greater number of HIV-infected patients survive despite profound immunodepression due to medical progress, we face complex infection with multiple agents in AIDS-patients.

**Case presentation:**

We report the case of an AIDS patient with a primary clinical presentation suggestive of bacillary angiomatosis. We also found in cutaneous lesions *Mycobacterium avium *complex and cytomegalovirus.

**Conclusion:**

This clinical case illustrates the possibility of multiple coinfections in AIDS patients and the need to be exhaustive in evaluating infectious diseases in severely immunocompromised patients.

## Background

As medical therapies for infection due to the human immunodeficiency virus (HIV) and its attendant complications improve, greater number of HIV-infected patients is surviving despite their profound immunodeficiency. *Bartonella quintana *and *Bartonella henselae *have been recognized as the causative agents of opportunistic infections, such as bacillary angiomatosis, peliosis hepatitis and bacteraemia, in patients with acquired immunodeficiency syndrome (AIDS). Although, *B. quintana*, historically the agent of trench fever, is usually transmitted by body lice, some patients have had no apparent louse exposure. Mycobacterial disease is a frequent cause of illness in AIDS patients with the majority of these patients having disseminated disease caused by *Mycobacterium avium *Complex (MAC). In profoundly immunocompromised patients, cytomegalovirus (CMV) also induced disseminated disease. These two agents rarely induce skin lesions in AIDS-patient. We report the clinical, microbiological and histopathologic findings of bacillary angiomatosis with concomitant infection by cytomegalovirus and *Mycobacterium *species in a patient with AIDS. This clinical case emphasizes the necessity to consider the possibility of concomitant infections in immunocompromised individuals and the necessity to look for multiple agents in skin biopsy specimens for such patients.

## Case presentation

A 51-year-old HIV-positive homosexual man, who has been followed at our hospital since 1992 for HIV, was admitted in June 2004 for persistent low-grade fever, night sweats and a 12 kilogram weight loss over one year. He was treated with antiretroviral therapy since 1995 and HAART since 1997. Unfortunately, the emergence of HIV variants with multiple resistance gene mutations resulted in a high HIV load and low CD4^+ ^T cell count. At the time of admission, the patient's CD4^+ ^T cell count was 8 cells/μl, and his HIV RNA level was 792,000 copies/ml. The body temperature ranged from 37°C to 38°5C. Physical examination revealed an enlarged liver and an extensive well-demarcated, violaceous plaque on the left ankle with 5 additional small violaceous nodules disseminated on the head, trunk and left leg, suggesting a diagnosis of bacillary angiomatosis (BA). A chest CT scan revealed a left lower-lobe density and sputum smears were positive for acid-fast bacilli (AFB). Pelvic CT scan revealed an inflammatory swelling within the right gluteus muscle that was biopsied as were the cutaneous lesion of the left ankle and the bone marrow.

## Microbiology and anatomopathology

**Figure 1:**
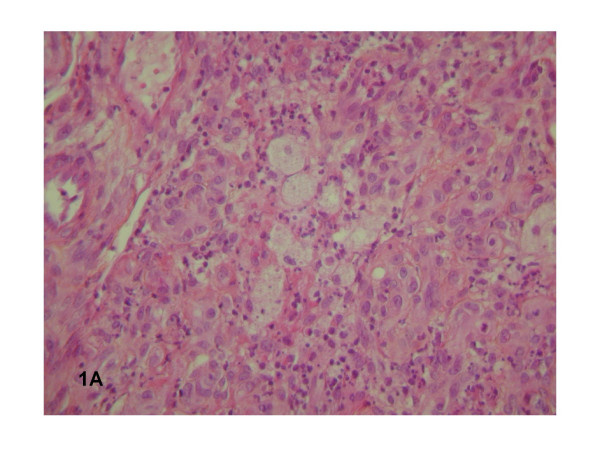
Histological examination of the skin biopsy sample demonstrating typical aspects of BA; clusters of foamy macrophages were also observed in the dermis, mixed with the histological features of BA.

**Figure 2:**
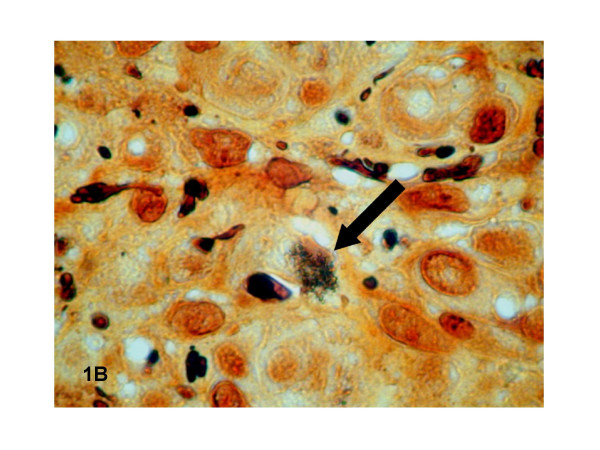
Clusters of bacteria (indicated by an arrow) were revealed on Warthin-Starry staining.

**Figure 3:**
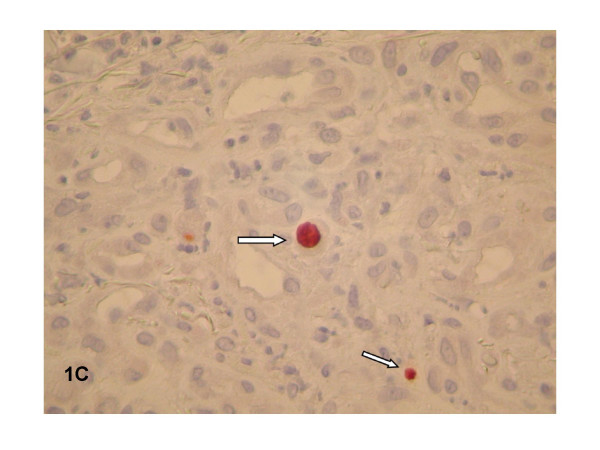
Immunohistochemical examination using anti-CMV monoclonal mouse antibody was positive (indicated by an arrow).

One half of the skin biopsy was inoculated onto Columbia sheep agar and human endothelial cells in shell vials for culture of *Bartonella *spp. and mycobacteria as previously described [1,2]. These procedures yielded isolation of two microorganisms that were identified as *Bartonella quintana *and *Mycobacterium avium *complex (MAC). Molecular detection of *B. quintana *using standard PCR targeting the 16S–23S intergenic spacer region [3] was positive for the cutaneous biopsy and had 100% homology with *B. quintana *strainFuller (Genbank accession number L35100). The other half of the skin biopsy was fixed in 5% formaldehyde, paraffin-embedded, sectioned to 4 μm in thickness, and stained with hematoxylin-eosin-saffron by use of routine methods. Serial sections were also obtained for special staining, including Warthin-Starry and Ziehl-Neelsen stains, or immunohistochemical investigations. Immunohistochemical analysis was performed using polyclonal rabbit antibodies (anti-*Bartonella henselae *and *B. Quintana*) or anti-CMV monoclonal mouse antibody (Clone E-13, Clonatec, Biosoft, Paris), diluted 1:500, 1:500 and 1:1000, respectively, in phosphate-buffered saline. The immunohistologic procedure, using an immunoperoxidase kit, has been described elsewhere [4]. Histological examination of the skin biopsy sample showed typical aspects of BA (Figure 1). A lobular capillary proliferation was visible in the dermis. The small vascular channels were lined with epithelioid endothelial cells that protruded into vascular lumens. An inflammatory infiltrate with numerous neutrophils was scattered throughout the lesion. Clusters of bacteria were revealed on Warthin-Starry staining (Figure 2) but immunohistochemical examination using the polyclonal rabbit antibodies anti-*B. henselae *and anti-*B. quintana *was negative. Moreover, clusters of foamy macrophages were also observed in the dermis, mixed with the histological features of BA. Tuberculous granulomas or necrotic areas were not observed, and the Ziehl-Neelsen stain was negative. We repeated Ziehl-Neelsen stain on the same biopsy fragment and it was still negative. CMV infection was detected in the skin biopsy specimen by characteristic intranuclear inclusion in proliferative endothelial cells and positive immunohistochemical examination using the anti-CMV monoclonal mouse antibody (Figure 3). Viral culture failed to identify CMV in the skin biopsy.

Cultures of the blood and bone marrow were also positive for *B. quintana*. Molecular detection of *B. quintana *using standard PCR targeting the 16S–23S intergenic spacer region [3] was positive for the gluteus muscle sample and had 100% homology with *B. quintana *strainFuller (Genbank accession number L35100). The sputum culture was positive for MAC, as were the broncho-alveolar liquid (BAL), blood culture and bone marrow. Histologic examination of the bone marrow revealed noncaseating granulomas.

The result of a CMV pp65 antigenemia assay, based on the direct detection of the CMV pp65 phosphoprotein was positive. PCR targeting of CMV immediate early antigen was also positive in heparinized blood sample.

Finally, evidence of *B. quintana *infection was detected in the cutaneous lesion, blood, bone marrow and gluteus muscle sample. Evidence of MAC infection was detected in the cutaneous lesion, blood, bone marrow and BAL. Evidence of CMV infection was detected in the cutaneous lesion, blood, and BAL.

## Treatment and outcome

Erythromycin (500 mg po qid) treatment was started upon result of the histology of the skin biopsy. Erythromycin was replaced by rifabutin (300 mg po qd), clarithromycin (500 mg po bid) and ethambutol (25 mg/kg po qd). After the first positive culture with MAC, the general state of the patient slightly improved but cutaneous lesions persisted. Kaposi's sarcoma was ruled out by another skin biopsy. Erythromycin susceptibility of the *Bartonella *strain was tested using an E-test derived assay [5] and the isolate was susceptible to erythromycin and gentamicin. The patient was followed every month clinically. The lesions finally disappeared with long term treatment with rifabutin, clarithromycin and ethambutol and general state improved. We did not prescribe anti-CMV treatment. The patient was still severely immunocompromised despite modifications of anti-retroviral treatment. One year later, he developped new cutaneous lesions. Anatomopathologic examination revealed Kaposi sarcoma. At the same time MAC infection relapsed after discontinuation of treatment (MAC isolated in blood culture).

## Discussion and conclusions

In this study we report the case of a male patient whose primary clinical presentation was suggestive of BA, but who was infected with four different agents at the same time. The four infections included HIV infection, BA with *B. quintana *isolation, MAC infection, and CMV infection. All four infections were eventually diagnosed unambiguously with several microbiological methods including culture, PCR, anatomopathology and immunodetection. The different methods used in this study have been previously validated for the diagnosis of these diseases and remain the reference techniques for such investigation.

Mycobacterial disease is a frequent cause of illness in AIDS patients with the majority of these patients having disseminated disease caused by MAC. Before the introduction of preventive antibiotic regimens, disseminated MAC disease was the most common clinical manifestation of MAC and the most common bacterial disease among patients with AIDS. Disseminated MAC occurs almost exclusively in patients with severe depression on CD4^+ ^cell count: the median CD4^+ ^cell count among patients with disseminated MAC and AIDS is 10 cells/mm^3^. Cutaneous disease due to MAC is uncommon and is not easily differentiated from other chronic skin lesions. Lesions may be ulcers, nodules, plaques, pustules or abscesses. When observed, cutaneous lesions are usually caused by dissemination of MAC; primary cutaneous infections with MAC are extremely rare. Although disseminated MAC infection is one of the most common infections identified in HIV-infected patients with fever and low CD4+ cell count, one study stated that more than one-half of the MAC culture-positive patients also had another cause of fever identified [6]. Rarely, coinfections with *Bartonella *spp. have been reported. Koehler *et al *[7] described 1% coinfection with *Bartonella *spp. and MAC in a series of 382 HIV patients but MAC was obtained solely from blood. To our knowledge, only 2 cases of bacillary angiomatosis and mycobacterium infection coexisting in a cutaneous lesion in patient with AIDS have been reported [8,9]. Clinically, lesions in all reported cases were consistent with a presomptive diagnosis of BA and a differential diagnosis of Kaposi's sarcoma. As for our patient, the lesion was characterized by capillary proliferation compatible with BA. Although Ziehl-Neelsen staining was negative, the presence of foamy macrophages in our patient suggested that MAC was also a possible etiologic agent responsible for the development of skin lesions [10]. Therefore, we can not assume that the skin lesion in our patient represents a lesion of BA with secondary involvement by *Mycobacterium avium *or whether the two organisms are implicated in the development of such lesions. In our case, the patient presented with BA and evidence of disseminated *Bartonella *infection since we retrieved either by culture or PCR, *B. quintana *in blood, cutaneous biopsy, gluteus muscle and bone marrow. The clinical significance of the presence of cytomegalovirus in cutaneous lesions of BA is unclear [11]. Cytomegalovirus skin lesions are rarely described in acquired immunodeficiency syndrome in contrast with the high frequency of ocular and visceral involvement [12]. To our knowledge, only one case of coinfection with *Bartonella, Mycobacterium avium *and CMV has been reported in a patient with AIDS [13]. Diagnosis was made by histology and immunohistochemistry but the different organisms could not be cultured. Another case reported the association of 3 organisms (*Staphylococcus aureus*, cytomegalovirus and acid-fast bacilli) in a single skin biopsy specimen [14].

We report here the clinical case of an AIDS patient with coinfection by *B. quintana*, MAC and CMV. All of them were retrieved in a skin biopsy for which anatomopathology was in favor of BA. It is therefore important to consider the possibility of complex infections in immunocompromised individuals and to look for multiple agents in skin biopsy specimens for such patients. This case emphasizes the need to be exhaustive when looking for opportunistic infections in severely immunodepressed AIDS patients.

## Competing interests

The author(s) declare that they have no competing interests.

## Authors' contributions

C Rovery participated to the conception of the study, participated to its coordination, drafted the paper and took in charge the patient in hospitalization. JM Rolain performed all bacteriologic assays (culture of *Bartonella *and *Mycobacterium avium*) and PCR assay for *Bartonella *and *Mycobacterium avium*. Hubert Lepidi performed anatomopathology and immunohistochemistry. Christine Zandotti performed all virologic studies (CMV viral culture and PCR assay). Jacques Moreau took in charge the patient, prescribed HAART and followed the patient. Philippe Brouqui conceived the study, coordinated it, finalized the paper and took in charge the patient in hospitalization. All authors read and approved the final manuscript.

## Pre-publication history

The pre-publication history for this paper can be accessed here:


